# Supplementary material for: Modeling glioblastoma heterogeneity as a dynamic network of cell states
Source: Mol Syst Biol. 2021 Sep 16;17(9):e10105. doi: 10.15252/msb.202010105 (PMC8444284; doi:10.15252/msb.202010105)
Supplement: Supplementary file 6 — Source Data for Figure 5 [file MSB-17-e10105-s004.zip › Figure5A_sourcedata/GSEA_3071/nosigdb_stateIII.GseaPreranked.1622795155562/IVY_CT.html]

Details for gene set IVY\_CT[GSEA]

|  || Dataset | state33071 |
| Phenotype | NoPhenotypeAvailable |
| Upregulated in class | na\_neg |
| GeneSet | IVY\_CT |
| Enrichment Score (ES) | -0.502845 |
| Normalized Enrichment Score (NES) | -3.106023 |
| Nominal p-value | 0.0 |
| FDR q-value | 0.0 |
| FWER p-Value | 0.0 |
Table: GSEA Results Summary

  

Fig 1: Enrichment plot: IVY\_CT      
 Profile of the Running ES Score & Positions of GeneSet Members on the Rank Ordered List

  

| PROBE | GENE SYMBOL | GENE\_TITLE | RANK IN GENE LIST | RANK METRIC SCORE | RUNNING ES | CORE ENRICHMENT || 1 | OIP5 |  |  | 537 | -0.256 | -0.1349 | No |
| 2 | NDC80 |  |  | 636 | -0.261 | -0.1552 | No |
| 3 | IGF2BP3 |  |  | 754 | -0.267 | -0.1803 | No |
| 4 | HES6 |  |  | 860 | -0.272 | -0.2022 | No |
| 5 | BCHE |  |  | 867 | -0.272 | -0.1983 | No |
| 6 | ID3 |  |  | 880 | -0.273 | -0.1959 | No |
| 7 | DHFR |  |  | 956 | -0.277 | -0.2099 | No |
| 8 | CDCA3 |  |  | 1029 | -0.281 | -0.2230 | No |
| 9 | RNASEH2A |  |  | 1103 | -0.285 | -0.2363 | No |
| 10 | SPATA6 |  |  | 1225 | -0.291 | -0.2620 | No |
| 11 | NUSAP1 |  |  | 1262 | -0.293 | -0.2654 | No |
| 12 | DTL |  |  | 1297 | -0.295 | -0.2684 | No |
| 13 | H19 |  |  | 1379 | -0.299 | -0.2835 | No |
| 14 | FADS2 |  |  | 1437 | -0.302 | -0.2922 | No |
| 15 | FIBIN |  |  | 1458 | -0.304 | -0.2913 | No |
| 16 | CREB5 |  |  | 1470 | -0.304 | -0.2880 | No |
| 17 | ETV1 |  |  | 1495 | -0.306 | -0.2881 | No |
| 18 | KIF4A |  |  | 1504 | -0.306 | -0.2840 | No |
| 19 | SLC10A4 |  |  | 1561 | -0.310 | -0.2924 | No |
| 20 | MYBL2 |  |  | 1587 | -0.311 | -0.2926 | No |
| 21 | SRPX |  |  | 1747 | -0.322 | -0.3276 | No |
| 22 | MEX3A |  |  | 1749 | -0.322 | -0.3214 | No |
| 23 | NUF2 |  |  | 1755 | -0.322 | -0.3162 | No |
| 24 | LFNG |  |  | 1859 | -0.327 | -0.3365 | No |
| 25 | HIST1H2AG |  |  | 1913 | -0.330 | -0.3436 | No |
| 26 | UBE2C |  |  | 1942 | -0.332 | -0.3442 | No |
| 27 | SALL1 |  |  | 1962 | -0.333 | -0.3425 | No |
| 28 | NMB |  |  | 1983 | -0.334 | -0.3409 | No |
| 29 | NLGN1 |  |  | 2107 | -0.342 | -0.3661 | No |
| 30 | SCD5 |  |  | 2128 | -0.344 | -0.3644 | No |
| 31 | KCNIP1 |  |  | 2131 | -0.344 | -0.3580 | No |
| 32 | RRM2 |  |  | 2147 | -0.345 | -0.3549 | No |
| 33 | DSEL |  |  | 2180 | -0.347 | -0.3563 | No |
| 34 | TPX2 |  |  | 2289 | -0.356 | -0.3773 | No |
| 35 | ASF1B |  |  | 2312 | -0.358 | -0.3758 | No |
| 36 | TCF12 |  |  | 2360 | -0.362 | -0.3807 | No |
| 37 | TTYH1 |  |  | 2397 | -0.365 | -0.3828 | No |
| 38 | DLGAP5 |  |  | 2421 | -0.367 | -0.3814 | No |
| 39 | ABAT |  |  | 2521 | -0.375 | -0.3996 | No |
| 40 | TACC3 |  |  | 2560 | -0.379 | -0.4019 | No |
| 41 | FAM111B |  |  | 2585 | -0.381 | -0.4005 | No |
| 42 | CDCA7 |  |  | 2710 | -0.391 | -0.4249 | No |
| 43 | SMC4 |  |  | 2872 | -0.405 | -0.4587 | No |
| 44 | SAPCD2 |  |  | 2894 | -0.407 | -0.4560 | No |
| 45 | ITGB8 |  |  | 3026 | -0.424 | -0.4816 | No |
| 46 | DCLK2 |  |  | 3039 | -0.425 | -0.4762 | No |
| 47 | SEMA6A |  |  | 3042 | -0.425 | -0.4682 | No |
| 48 | CCNE2 |  |  | 3138 | -0.436 | -0.4841 | No |
| 49 | SOX11 |  |  | 3205 | -0.443 | -0.4924 | No |
| 50 | CDCA2 |  |  | 3212 | -0.444 | -0.4850 | No |
| 51 | PCDHGC3 |  |  | 3213 | -0.444 | -0.4761 | No |
| 52 | HIST1H2AH |  |  | 3216 | -0.445 | -0.4676 | No |
| 53 | SOX21 |  |  | 3352 | -0.471 | -0.4933 | Yes |
| 54 | METRN |  |  | 3358 | -0.472 | -0.4851 | Yes |
| 55 | PCBP4 |  |  | 3381 | -0.476 | -0.4813 | Yes |
| 56 | KIF14 |  |  | 3384 | -0.476 | -0.4722 | Yes |
| 57 | EGFR |  |  | 3433 | -0.488 | -0.4748 | Yes |
| 58 | NPAS3 |  |  | 3434 | -0.488 | -0.4650 | Yes |
| 59 | GTSE1 |  |  | 3468 | -0.498 | -0.4636 | Yes |
| 60 | OLIG1 |  |  | 3486 | -0.503 | -0.4579 | Yes |
| 61 | GAS1 |  |  | 3494 | -0.505 | -0.4495 | Yes |
| 62 | NCAPG |  |  | 3513 | -0.512 | -0.4439 | Yes |
| 63 | SOX9 |  |  | 3560 | -0.531 | -0.4451 | Yes |
| 64 | CKB |  |  | 3572 | -0.536 | -0.4372 | Yes |
| 65 | LRRC17 |  |  | 3590 | -0.543 | -0.4307 | Yes |
| 66 | NES |  |  | 3596 | -0.545 | -0.4210 | Yes |
| 67 | SOX2 |  |  | 3613 | -0.555 | -0.4140 | Yes |
| 68 | HIST1H1D |  |  | 3637 | -0.569 | -0.4085 | Yes |
| 69 | MATN2 |  |  | 3641 | -0.572 | -0.3977 | Yes |
| 70 | MLC1 |  |  | 3659 | -0.584 | -0.3904 | Yes |
| 71 | TOP2A |  |  | 3671 | -0.594 | -0.3813 | Yes |
| 72 | GPR37 |  |  | 3691 | -0.609 | -0.3740 | Yes |
| 73 | CENPE |  |  | 3711 | -0.625 | -0.3663 | Yes |
| 74 | VCAN |  |  | 3741 | -0.660 | -0.3606 | Yes |
| 75 | HIST1H1B |  |  | 3749 | -0.669 | -0.3489 | Yes |
| 76 | KLHDC8A |  |  | 3750 | -0.672 | -0.3353 | Yes |
| 77 | FAM181B |  |  | 3760 | -0.686 | -0.3238 | Yes |
| 78 | SOX8 |  |  | 3797 | -0.737 | -0.3184 | Yes |
| 79 | FREM2 |  |  | 3807 | -0.755 | -0.3055 | Yes |
| 80 | NCAN |  |  | 3813 | -0.761 | -0.2914 | Yes |
| 81 | LRRN1 |  |  | 3816 | -0.766 | -0.2765 | Yes |
| 82 | KCNQ2 |  |  | 3826 | -0.787 | -0.2630 | Yes |
| 83 | WSCD1 |  |  | 3835 | -0.798 | -0.2490 | Yes |
| 84 | CENPF |  |  | 3842 | -0.820 | -0.2340 | Yes |
| 85 | ASCL1 |  |  | 3852 | -0.843 | -0.2193 | Yes |
| 86 | LRRN3 |  |  | 3859 | -0.856 | -0.2036 | Yes |
| 87 | SCRG1 |  |  | 3869 | -0.904 | -0.1877 | Yes |
| 88 | DLL3 |  |  | 3873 | -0.948 | -0.1694 | Yes |
| 89 | POU3F2 |  |  | 3875 | -0.953 | -0.1504 | Yes |
| 90 | C1orf61 |  |  | 3879 | -0.986 | -0.1313 | Yes |
| 91 | PTN |  |  | 3884 | -1.022 | -0.1117 | Yes |
| 92 | ID4 |  |  | 3893 | -1.061 | -0.0924 | Yes |
| 93 | COL9A3 |  |  | 3896 | -1.084 | -0.0711 | Yes |
| 94 | ASPM |  |  | 3899 | -1.166 | -0.0481 | Yes |
| 95 | PTPRZ1 |  |  | 3913 | -1.342 | -0.0244 | Yes |
| 96 | IGFBP2 |  |  | 3917 | -1.404 | 0.0031 | Yes |
Table: GSEA details [plain text format]

  

Fig 2: IVY\_CT: Random ES distribution      
 Gene set null distribution of ES for **IVY\_CT**
